# Transcriptomic Changes Induced by Low and High Concentrations of Heavy Metal Exposure in *Ulva pertusa*

**DOI:** 10.3390/toxics11070549

**Published:** 2023-06-22

**Authors:** Do Yeon Seo, Mira Park, Jeong-In Park, Jang K. Kim, Seungshic Yum, Youn-Jung Kim

**Affiliations:** 1 Risk Assessment Division, Environmental Health Research Department, National Institute of Environmental Research, Incheon 22689, Republic of Korea; dyseo0822@korea.kr; 2 Department of Marine Sciences, Incheon National University, Incheon 22012, Republic of Korea; qkrwjdls0812@naver.com (J.-I.P.); jang.kim@inu.ac.kr (J.K.K.); 3 Research Institute of Basic Sciences, Incheon National University, Incheon 22012, Republic of Korea; mira0295@inu.ac.kr; 4 Ecological Risk Research Division, Korea Institute of Ocean Science and Technology (KIOST), Geoje 53201, Republic of Korea

**Keywords:** marine protection standards (MPS), heavy metals, copper (Cu), cadmium (Cd), RNA sequencing, *Ulva pertusa* (*U. pertusa*)

## Abstract

The impact of sewage and wastewater pollution on marine ecosystems is of increasing concern due to the rapid accumulation of heavy metals in seaweeds inhabiting near-shore environments. Seaweeds can be severely damaged by heavy metals throughout their life cycles. Although the physiological and ecological effects of heavy metal exposure have been studied, there is limited research on their molecular responses. *Ulva pertusa* is a prevalent seaweed species in South Korea and is ecologically significant in coastal ecosystems. We utilized high-throughput RNA sequencing to analyze changes in the transcriptome profiles of *U. pertusa* under low concentrations of heavy metals (MPS) and high concentrations of copper (MPS-Cu) and cadmium (MPS-Cd). Differential gene expression analysis revealed that 53 (control vs. MPS), 27 (MPS vs. MPS-Cd), and 725 (MPS vs. MPS-Cu) genes were expressed differentially. Differentially expressed genes identified in our study included those with protective roles against oxidative stress and those involved in metal transport to the vacuole. Furthermore, exposure to heavy metal stress had a negative impact on the photosynthetic apparatus structural proteins of *U. pertusa*, resulting in photosynthetic inhibition. Moreover, exposure to high concentrations of copper resulted in the activation of carbon-related metabolism. These findings contribute to our understanding of the molecular mechanisms underlying heavy metal toxicity in *U. pertusa*.

## 1. Introduction

Environmental issues such as global warming, ocean acidification, and water pollution due to industrial and domestic sewage have garnered significant attention in marine ecosystems. These concerns have become a worldwide issue, with heavy metal pollutants derived from anthropogenic sources, such as waste, urban effluents, and domestic waste, severely affecting coastal areas [1]. In particular, seaweeds efficiently absorb and accumulate heavy metals from seawater [2,3,4,5]. Researchers have raised concerns that such bioaccumulation in primary producers can affect primary and secondary consumers, thereby destroying the coastal ecosystem [6].

Seaweeds play a critical role as primary producers in coastal ecosystems, and their exposure to heavy metal pollutants can have negative impacts on the entire food chain, affecting the health of consumers such as fish and invertebrates [6]. *Ulva pertusa* is a dominant seaweed species that is widespread along the coast of Korea and is particularly vulnerable to heavy metal pollution due to its proximity to land-derived sources of pollutants. Since the 19th century, heavy metal pollution caused by rapid industrialization has become a major environmental issue in coastal ecosystems.

Some heavy metals, such as iron, manganese, copper, and zinc, are essential for algae. Seaweeds can maintain metal homeostasis at proper levels, but their exposure to heavy metals above baseline levels may have toxic effects on algal metabolism [7,8,9]. Copper, for instance, is crucial for algae and is present in plastocyanin, a copper-containing protein that plays a key role in photosynthesis [10]. However, heavy metal-induced toxic effects can cause changes in cell size and morphology, resulting in serious damage to the growth and physiological processes of algae [11]. 

When seaweeds are exposed to high concentrations of heavy metals, the metal ion concentration inside the cytoplasm is lower than it is outside the cell. Owing to the concentration difference, metal ions flow into the cell through the cell membrane. The entry of metal ions into the cytoplasm generates free oxygen, which can produce reactive oxygen species that damage cells through oxidative stress [12]. Studies have shown that high copper concentrations can lead to biological damage in both physiological and molecular responses [13]. Cadmium exposure also affects photosynthesis, leading to a decrease in photosynthetic capacity, efficiency, and productivity in plants [14]. Several studies on the effects of heavy metals in *Ulva* have reported a decrease in photosynthetic capacity, efficiency, and productivity [15,16,17]. Thus, seaweeds regulate the levels of metal ions in the cytoplasm through various mechanisms to resist heavy metal toxicity. Seaweeds may express specific genes associated with heavy metal toxicity resistance mechanisms. Previous studies on the impact of heavy metal pollutants on seaweeds have primarily focused on heavy metal accumulation and adsorption by seaweeds [5,6]. However, recent advancements in sequencing technology have enabled the generation of genetic information for various seaweed species. Studies have reported identifying genes that alter under environmental stress conditions such as light, salinity, heavy metal exposure, etc. Studies have also explored the underlying mechanisms of action of these stress responsive genes in seaweeds. Some of the seaweeds that have been studied at the molecular level include Ulvaceae such as *U. prolifera* [18,19], *U. linza* [20], and *U. compressa* [21,22].

*U. pertusa* is a cosmopolitan species and one of the ecologically important seaweed species [23]. Nevertheless, the precise molecular mechanisms that underpin metal tolerance in seaweed remain elusive. A major obstacle to understanding the stress responses of heavy metal in seaweeds is the lack of genetic information. 

In the present study, we utilized high-throughput RNA sequencing to investigate the changes in transcriptome profiles of *U. pertusa* when exposed to low concentrations of heavy metals conforming to the marine life protection standard (MPS) and when exposed to high concentrations of heavy metals (cadmium or copper) that induce significant physiological changes. *U. pertusa* subjected to various types of heavy metal exposure conditions will induce expression changes in a greater variety of genes than the control group. Since this study is the first sequencing of mRNA of *U. pertusa*, this strategy was used to effectively obtain sequence information of more kinds of genes. Genes differentially expressed in response to heavy metal exposure were identified and functionally characterized. Additionally, the implications of cadmium and copper metal toxicity mechanisms on photosynthetic activity by measuring the Electron Transport Rate (ETR, converted into chemical energy during photosynthesis), Maximum PSII quantum yield (Fv/Fm measures the potential efficiency of light energy conversion in the photosynthetic system), Maximum Electron Transport Rate (ETRmax), photosynthetic efficiency (α), and light saturation points of photosynthesis (Ek, the minimum saturation irradiance) were evaluated. The RNA sequencing data gathered from this study could provide insights into the defense and adaptation strategies of *U. pertusa* against heavy metals across various stress conditions. Moreover, this study aims to identify specific genes associated with heavy metal resistance and contribute to the development of a comprehensive genetic information profile of *U. pertusa*.

## 2. Materials and Methods

### 2.1. Seawater Sampling and Cultivation Conditions

*U. pertusa* was collected from the intertidal zones of Gunnae-ri, Wando-eup, Wando-gun, and Jeollanam-do. The collected samples were kept in a cooler and transported to the laboratory within 5 h. Upon arrival, the samples were washed multiple times with sterilized artificial seawater, before being transferred to a 20 L plastic container containing the same seawater medium. The culture medium was prepared by adding artificial salt (Coralife Energy Savers, Central Garden & Pet, Walnut Creek CA, USA) to distilled water to adjust the salinity to 35‰, and 1 mM of KNO_3_ and 0.1 mM of K_2_HPO_4_ (Sigma-Aldrich, St. Louis, MO, USA) were also added to the medium. *U. pertusa* cultures were aerated in sterilized artificial seawater at 15 °C under a 12:12 h L:D photoperiod provided by white fluorescent bulbs (FL400; Kumho Electric Inc., Seoul, Republic of Korea). Prior to the experiment, the cultures were acclimated for at least three days under the same conditions. 

### 2.2. 2,3,5-Triphenyltetrazolium Chloride (TTC) Reduction Assay

The effects of heavy metals on viability were evaluated using a 2,3,5-Triphenyltetrazolium chloride (TTC) reduction assay. We followed a modified version of a previous protocol [24,25]. *U. pertusa* thallus was cut into circular slices using a 9 mm diameter cork borer. Using a 24-well plate, one tissue sample was placed in each well and treated with 1 mL of heavy metal solution at each concentration. The concentrations of the treated copper (Cu) solutions were 0, 7.8125, 15.625, 31.25, 62.5, 125, and 250 µg/L in sterilized artificial seawater. The concentrations of the treated cadmium (Cd) solutions were 0, 313, 625, 1250, 2500, and 5000 µg/L in sterilized artificial seawater. The samples were then cultured for 72 h under the conditions described above and rinsed with distilled water after exposure to the heavy metals. Then, the samples were immersed in an incubation solution (50 mM sodium phosphate, pH 7.4) containing 0.8% TTC solution and incubated for 18 h at 15 °C in the dark. After extraction with 95% ethanol twice, the extracts were combined and adjusted to a 1 mL volume. The resulting ethanol extract was transferred to a 96-well plate, and the formazan formed in the green tissues was measured at 530 nm instead of 485 nm to minimize interference from pigments such as chlorophyll [26].

### 2.3. Sterilization

To prevent the algal tissue from microbial contamination, the tissue was surface-sterilized by treating it for 30 sec with 0.5% hypochlorous acid (HOCl; Sigma-Aldrich, St. Louis, MO, USA) [20,27]. After the HOCl treatment, the tissue was rinsed with distilled water three times for 5 min to remove residual reagents.

### 2.4. Chemical Treatment (Marine Protected Standard (MPS), MPS-Cd, and MPS-Cu Treatment)

The transcriptomic changes caused by heavy metal stress in *U. pertusa* were analyzed in two different ways: (1) *U. pertusa* exposed to the heavy metals of low concentration equivalent to marine protection standard (MPS; 3.0 µg/L of copper, 19 µg/L of cadmium, 200 µg/L of chromium 6^+^, and 11 µg/L of nickel), compared to without heavy metal stress condition (Control). (2) *U. pertusa* exposed to high concentrations of heavy metals (e.g., additional 10,000 µg/L of cadmium (MPS-Cd), additional 250 µg/L of copper (MPS-Cu)) after exposure to low concentrations of heavy metals (MPS). The method of treatment of heavy metals in *U. pertusa* is briefly drawn, as shown in Figure S1. Sterilized *U. pertusa* (0.2 g) was exposed to 15 mL of aqueous solution containing heavy metals in a 100 × 20 mm Petri dish (SPL Life Sciences, Pyeongtaek, Gyeonggi-do, Republic of Korea) at each experimental concentration. Based on the concentration of heavy metals corresponding to the MPS (November, 2016) proposed by the Ministry of Oceans and Fisheries (MOF) of South Korea, concentrations corresponding to the short-term concentrations for each chemical were used in the experiment (Table S1). The concentration of each heavy metal was 3 µg/L of copper, 19 µg/L of cadmium, 11 µg/L of nickel, and 200 µg/L of chromium 6^+^, respectively. For the treatment of heavy metals, 1000 µg/L standard solution (Junsei Chemical Co., Ltd., Nihonbashi-honcho, Chuo-ku, Tokyo, Japan) was used. Other culture conditions were the same as those mentioned above for *U. pertusa* [28]. The *U. pertusa* samples of all groups were incubated for three days in a medium not treated with heavy metals for the stabilization. The medium containing the heavy metals was not replaced during the test period. The *U. pertusa* samples were subjected to low concentrations of heavy metals for a period of three days, following which they were rinsed with sterilized seawater. Subsequently, the samples were exposed to high concentrations of copper or cadmium (250 µg/L of Cu and 10,000 µg/L of Cd) for a further three days. Media containing chemicals were removed from the surface of *Ulva* as much as possible using a paper towel, and the samples were then immediately frozen using liquid nitrogen to facilitate RNA extraction, and then stored in a −80 °C deep freezer. All the experimental samples were prepared in five replicates. 

### 2.5. Photosynthesis and Chlorophyll Fluorescence Measurement

To assess photosynthetic activity, we selected samples from the Control, MPS, MPS-Cu-, and MPS-Cd-treated *U. pertusa* patches. Five replicates from each treatment were chosen, and the rapid light curve (RLC) was determined using a Mini-PAM II fluorometer with a 2035-B leaf-clip holder (Walz, Effeltrich, Germany). The RLC represents the saturation characteristics of PSII electron transport and overall photosynthetic performance, with each exposure lasting 20 s. For RLC production, the samples were exposed to 12 intensities of actinic light (0, 25, 65, 91, 125, 191, 288, 422, 632, 823, and 1150 μmol photons m^−2^ s^−1^). For each level of actinic light, the electron transport rate (ETR) was estimated as PSII, Φ_P_ = ΔF/F_m_′ = (F_m_′ − F)/F_m_′, and the photosynthetic photon flux density (PPFD), where the effective quantum yield (PSII) is the effective photochemical quantum yield of Photosystem II. PPFD is the value recorded by the light sensor in the leaf clip, 0.84 is the estimated mean proportion of incident light absorbed by the photosystems [29], and 0.5 accounts for the photons absorbed by both photosystems [30]. The light response was characterized by fitting the model of Platt et al. [31] to the ETR versus PPFD curves and by estimating the initial slope of photosynthetic efficiency (α), maximum electron transport rate (ETRmax), and half-saturating irradiance (E_k_). The maximum PSII quantum yield was calculated as F_v_/F_m_ = (F_m_ − F_0_)/Fm. The total chlorophyll content of each patch was measured using a CCM-300 Chlorophyll Content Meter (Opti-Sciences Inc., Hudson, NH, USA).

### 2.6. RNA Extraction

The frozen samples were transferred directly to a mortar and ground into powder using a pestle while continuously adding liquid nitrogen to keep the samples frozen, and total RNA was isolated from each sample using RNAiso Plus (Takara, Minato-ku, Tokyo, Japan) following the manufacturer’s instructions. RNA concentration and purity were determined using a Nanodrop LITE spectrophotometer (Thermo Fisher Scientific, Waltham, MA, USA). The RNA integrity number (RIN) of the total RNA samples was measured using an Agilent 2100 BioAnalyzer (Agilent Technologies, Santa Clara, CA, USA). A composite RNA sample pooled from five replicates per treatment was used for RNA-seq.

### 2.7. Sequencing Library Preparation and RNA-Seq

To obtain gene expression information, total RNA samples from *U. pertusa* exposed to different heavy metal conditions were pooled for Illumina sequencing. mRNA was prepared from 2 µg of total RNA extracted from each sample in the previous procedure using oligo (dT) magnetic beads. Fragmented mRNAs were synthesized as single-stranded cDNA through random hexamer priming. The libraries were performed to 100 bp paired-end sequencing using the TruSeq RNA Sample Preparation Kit (Illumina, San Diego, CA, USA). They were quantified using the KAPA library quantification kit (Kapa Biosystems, Wilmington, MA, USA), according to the manufacturer’s library quantification protocol. Following cluster amplification of the denatured templates, sequencing was conducted as a paired-end base using an Illumina HiSeq2500 (Illumina, San Diego, CA, USA). All reads were deposited in the Short Read Archive (SRA) of the National Center for Biotechnology Information (NCBI) with accession number PRJNA604909.

### 2.8. De Novo Assembly and Annotation

Prior to assembly, filtering was performed to remove low-quality and adapter sequences according to the following criteria: reads containing more than 10% skipped bases, reads containing more than 40% bases whose quality scores were less than 20, and reads with average quality scores of less than 20. Additionally, the bases of both ends that were less than Q20 of the filtered reads were removed. This process is to enhance the quality of reads due to mRNA degradation in both ends of it as time elapses [32]. Transcriptome assembly was performed using the Trinity [33,34] assembler, using data from all samples. The assembled transcriptome was grouped using TGICL [23,35]. To predict the function of the unigenes, we predicted the protein expression region (coding sequence, CDS) as a preliminary step. This process was performed using TransDecoder [34]. BLAST and InterProScan were used for homology search to predict the function of CDS in unigenes. NCBI BLAST 2.2.28+ was used for nucleotide sequence-based homology search. The criterion for the significance of similarity was set to an *E*-value < 0.00001. Differentially Expressed Genes (DEGs) were identified based on a *q*-value threshold less than 0.05.

### 2.9. Differential Gene Expression Analysis

The gene expression levels in each sample were measured using RSEM [36]. RSEM is a tool for measuring the expression levels of genes without reference to genome information. The TCC package was used for DEG analysis through the iterative DEGES/DEseq method. This method is based on DESeq [37], using a negative-binomial distribution. Selected genes with *p*-values < 0.05 and Fold change >2 following the test were regarded as statistically significant. Compared with the comparison group (Control or MPS), genes with *p*-value < 0.05 and Fold change >2 were selected as DEGs, and further analysis was carried out.

### 2.10. Gene Ontology (GO) and Kyoto Encyclopedia of Genes and Genomes (KEGG) Analysis

The gene Ontology (GO) database classifies genes according to the three GO terms of Biological Process (BP), Cellular Component (CC), and Molecular Function (MF), and it provides information on the function of genes. To characterize the identified genes from the DEG analysis, a GO-based trend test was carried out using Fisher’s exact test [38]. All DEGs were mapped to the gene symbol in the KEGG database [39] and compared with the whole transcriptome background to search for genes involved in significant heavy metal stress-related pathways. 

### 2.11. RT-qPCR for Gene Expression Analysis

Approximately 500 ng of total RNA was converted into cDNA using ReverTra Ace^®^ qPCR RT Master Mix with a gDNA remover (Toyobo, Osaka, Japan). The quantitative amplification reaction was performed on a CFX Connect Real-Time System (Bio-Rad, Hercules, CA, USA) using the THUNDERBIRD SYBR qPCR Mix (Toyobo, Osaka, Japan). The reaction mixture (15 μL) contained 2× Power SYBR Green mixture, 0.5 pmol each of the forward and reverse primers, 1 μL of template cDNA, and the remaining volume was DEPC water. PCR amplification was performed under the following conditions: initial activation step for 3 min at 95 °C, 3-step amplification cycling; denaturation for 10 s at 95 °C, annealing for 30 s at the corresponding temperature for each primer; extension for 30 s at 72 °C; and cycle number of 40 cycles. Primers used for qPCR are listed in Table S2. The expression of target genes was normalized to that of the reference gene (Histone *H2AX*) and the normalized expression was calculated using the Cq values and amplification efficiencies of Histone *H2AX* and the target genes (Tables S2 and S3). Gene-specific primer pairs were designed using Primer3 (version 4.0.0). RefFinder was utilized to determine the stable reference gene rankings based on the results from the Delta CT, BestKeeper, Normfinder, and Genorm programs.

### 2.12. Statistical Analysis

Statistical significance was tested using one-way analysis of variance (ANOVA) and *post hoc* Tukey’s HSD test in GraphPad Prism 5 software (the significance levels were set to 0.05, 0.01, or 0.001; * *p*  <  0.05, ** *p* < 0.01, *** *p*  <  0.001, ns: not significant or *p*  >  0.05). Pearson’s correlation coefficient (*r*) was used to measure the strength of linear associations between parameters.

## 3. Results

### 3.1. Effects on Morphology and TTC Reduction Ability of U. pertusa via Heavy Metal Treatment

**Figure 1:**
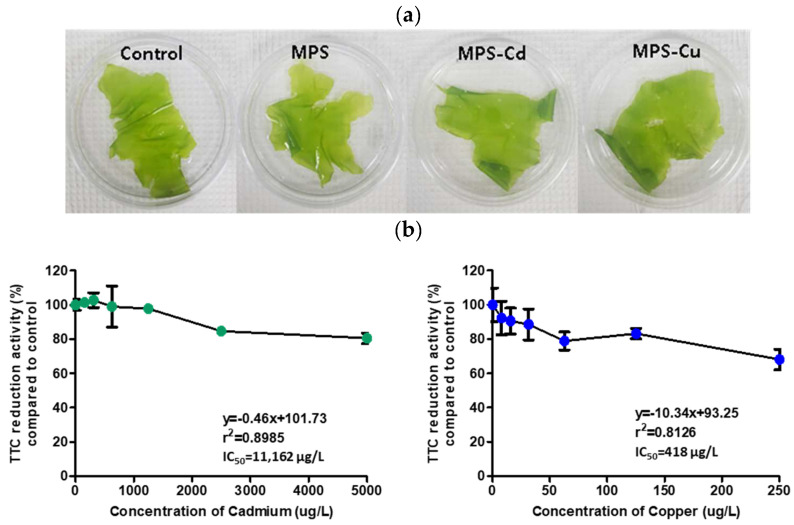
Assessment of heavy metal toxicity. (**a**) Morphology of *U. pertusa* tissues, (**b**) *U. pertusa* viability by TTC assay. *U. pertusa* tissues were placed into 24-well plates and subjected to different concentrations of heavy metals. After 72 h of incubation, 0.8% TTC solution was added to each plate, and the cells were incubated at 15 °C for 18 h in darkness. TTC reduction activity is expressed as a percentage of absorbance on treated groups with respect to the control. The results are expressed as mean ± standard deviation of three independent experiments.

We examined the change of morphology and viability of *U. pertusa* via heavy metal treatments for 72 h. There were no discernible differences in morphology between the control and heavy metal-treated groups (Figure 1a). The respiratory dehydrogenase-dependent TTC reduction ability of *U. pertusa* to cadmium and copper exposure was evaluated after three days of exposure to various heavy metal concentrations. A dose-dependent TTC reduction was observed in both cadmium- and copper-treated *U. pertusa* tissues (Figure 1b). The 50% inhibition concentration (IC_50_) values obtained were 11,162 µg/L for cadmium and 418 µg/L for copper. For the preparation of MPS-Cd and MPS-Cu samples, 10,000 µg/L of Cd and 250 µg/L of Cu were treated at concentrations that maintained 70% viability in *U. pertusa*.

### 3.2. An Overview of Transcriptome Sequencing and De Novo Assembly

To obtain a general overview of the transcriptome profiles of *U. pertusa* and to identify genes involved in heavy metal responses, RNAs obtained from four different conditions (Control, MPS, MPS-Cd and MPS-Cu) were sequenced using the Illumina HiSeq2500 platform. Transcriptome sequencing generated 262,697,020 raw reads in total: 61,713,540 (control), 62,244,430 (MPS), 67,608,248 (MPS-Cd), and 71,130,802 (MPS-Cu) raw reads. Around 90.1–91.3% of the initial raw reads were retained after the read trimming and filtering processes (Table S4). Contigs were assembled into unigenes through clustering using the TGICL program. The 86,039 transcripts represented 60,909 genes, with an average length of 941 bp, N50 of 1594 bp, and an average GC content of 54.5% (Table 1). The length distribution of the assembled transcripts is shown in Figure S2. 

### 3.3. Annotation and Functional Classification of Transcripts

**Figure 2:**
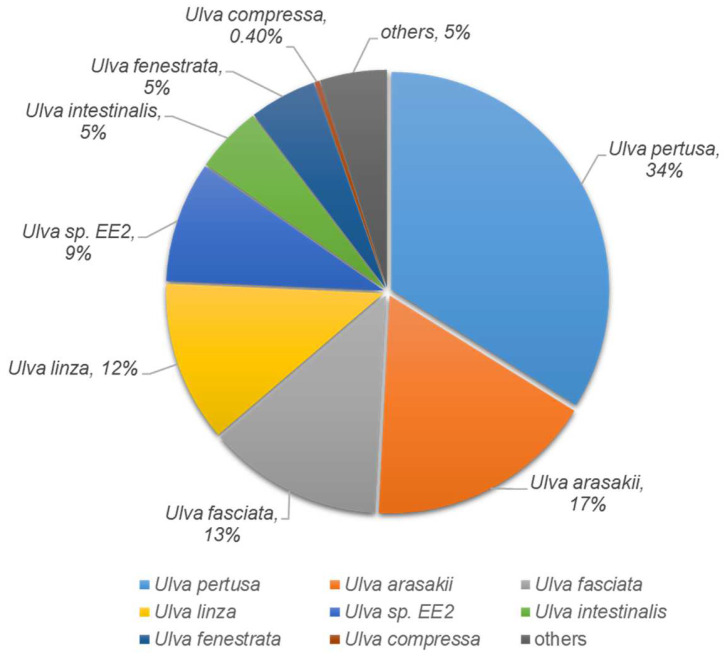
The Pie chart shows the species distribution of the top BLASTX hits for homologous sequences.

To identify the biological responses of *U. pertusa* to heavy metal exposure, the known and predicted functions of the heavy metal stress-responsive transcripts were investigated using InterProScan with an e-value cutoff of 10^−5^. A total of 17,724 unigenes (29.1%) yielded significant BLASTX results. The distribution of the top-hit species from the BLASTX matches is shown in Figure S3A. The majority of the sequences were best matched with sequences from the plant group (55%), followed by those from bacteria (11%) and invertebrates (8%). The remaining 26% were related to primate, rodent, and vertebrate sequences. A total of 20,332 unigenes (33.2%) yielded significant InterProScan results. The remaining 41,001 (66.8%) unigenes did not show any significant results (Figure S3B). A total of 15,142 unigenes (24.9%) were jointly matched in both the programs. Only 2582 unigenes (4.2%) and 2657 unigenes (4.4%) were uniquely matched in Blastx and InterProScan, respectively. The remaining 40,528 unigenes (67%) did not show homology with the deposited sequences used in either of the search tools (Figure S3C). Based on these results, one or more genes in BLASTX and InterProScan were used in subsequent analyses. *U. pertusa* taxonomy from vertical profiling was imported and displayed using the Krona metagenome visualizer. The taxonomy nodes are presented as nested sectors, arranged from the center outward, with the top level of the hierarchy at the center. Navigation controls are located at the top left, while details of the selected node are displayed at the top right [40]. Protein matches in other plant species were found to 9795 (55%) via BLASTX analysis. The species distribution showed that 20% of the isolates had top matches with Chlamydomonas reinhardtii. However, the average e-value criterion had the best hits with *U. pertusa*, the species closest to Ulvophyceae, as expected (Figure S4). To investigate the species specificity of these unigenes, we matched them against the NCBI-nr database. Of these unigenes, 34% were most similar to sequences of *U. pertusa*, and 17% and 13% of unigenes showed high similarity to sequences of *U. arasakii* and *U. fasciata*, respectively (Figure 2). 

### 3.4. Identification of Differentially Expressed Genes (DEGs)

**Figure 3:**
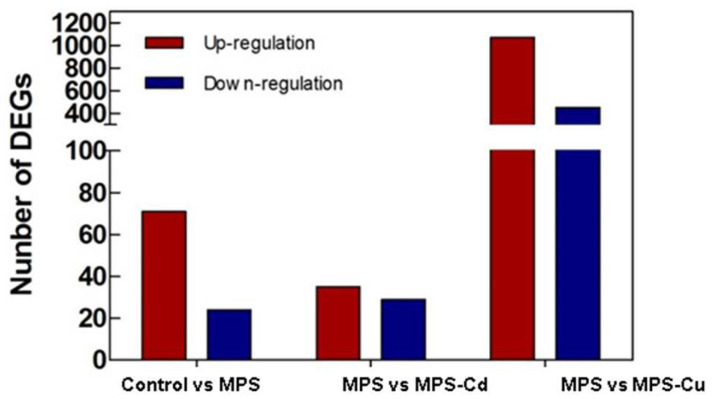
Differentially expressed genes of *U. pertusa* in response to heavy metal stress. Number of significantly upregulated and downregulated genes are displayed in red and blue bars, respectively (*p*-value of false discovery rate ≤0.05 and absolute value of fold change >2).

Using log2-transformed Fragments Per Kilobase of transcripts per Million fragments mapped (FPKM) data, we examined the correlations between pairs of samples using Pearson correlation coefficients. The Pearson correlation coefficient was r = 0.854 in the control vs. MPS-Cu, r = 0.752 in the MPS-Cd vs. MPS, and r = 0.781 in the control vs. MPS groups (Figure S5). The significant difference analysis was based on the normalized FPKM value determined using an RSEM-based algorithm. All differentially expressed genes (DEGs) with *p*-value ≤ 0.05 and fold change > 2 were identified between each pair of samples. Seventy-one genes were upregulated and 28 genes were downregulated via MPS treatment compared to control. After MPS-Cd treatment, 64 genes showed a difference in expression compared to that of the control. Of these, 35 genes showed increased expression and 29 genes showed decreased expression. In addition, in the MPS-Cu treatment group, 1552 genes showed different expression levels compared to those of the MPS group, 1088 genes showed increased expression, and 464 genes showed decreased expression (Figure 3). These results showed that the impact of Cu-treatment on *U. pertusa* transcriptome was larger than that of Cd treatment and low levels of heavy metal treatments.

### 3.5. Gene Ontology

**Figure 4:**
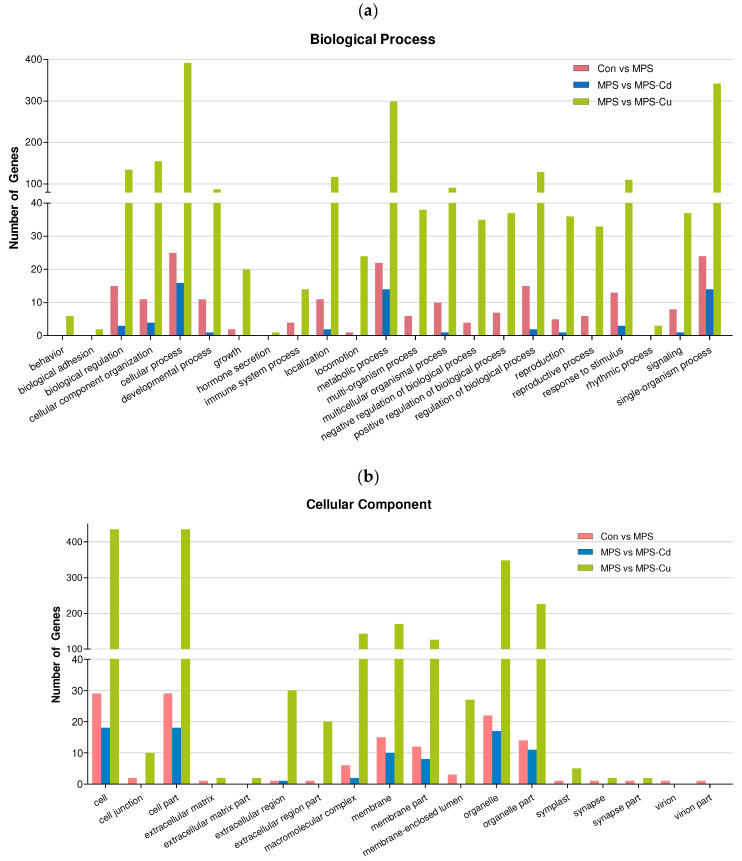

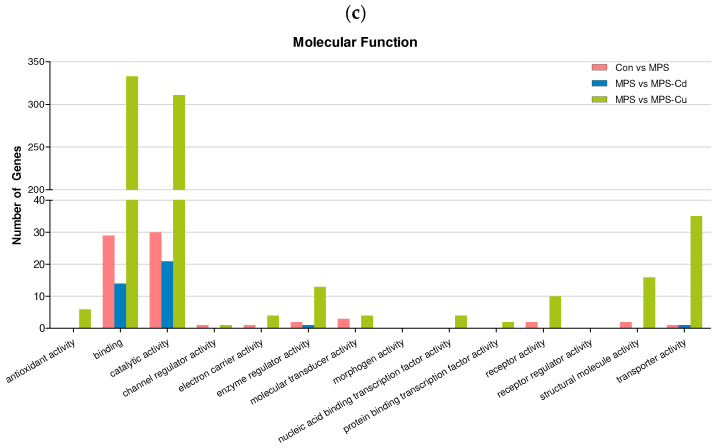
Gene ontology (GO) classification of DEGs for Control vs. MPS (pink); MPS vs. MPS-Cd (blue); MPS vs. MPS-Cu (green). GO terms were annotated at the level 2 categories, including biological process (**a**), cellular component (**b**) and molecular function (**c**).

Gene ontology (GO) analysis was conducted to further investigate the characteristics of the genes selected through differential gene expression analysis. Based on these DEGs, 1610 GO terms were assigned to 636 DEGs. The distribution of GO terms for the three main categories (biological processes, cellular components, and molecular functions) is shown in Figure 4. The results from the GO analysis showed that the genes involved in biological processes were the most abundant, and that the number of genes involved in cellular components was relatively small. 

Figure 4a shows the results of the Gene Ontology (GO) analysis for “biological processes”. DEGs in the “Control vs. MPS group” were divided into 142 subcategories. Among these, the majority of genes were related to the “cellular process” (47.2%), “single-organism” (45.3%), and “metabolic process” (41.5%) functions. When comparing MPS with MPS-Cd, the DEGs were grouped into 26 subcategories and were primarily related to the functions of the “cellular process” (59.3%), “metabolic process” (51.9%), and “single-organism” (51.9%), but the response to “cellular component organization or biogenesis” was only 14.8%. In the MPS vs. MPS-Cu group, DEGs were divided into 762 subcategories, the most frequently matched genes were related to “cellular process” (53.8%), followed by “single organisms” (46.9%) and “metabolic processes” (41.4%). Through these results, it can be observed that the DEGs affected by heavy metal exposure are involved in the biological processes of “cellular process”, “single-organism”, and “metabolic process”. Particularly, a greater diversity and a larger number of subcategories of biological process were observed in the MPS vs. MPS-Cu group. In the case of the MPS vs. MPS-Cd group, it was observed that the biological process categories altered in the con vs. MPS group were mostly further increased. However, in the case of MPS vs. MPS-Cu group, it was noticeable that a significantly larger number of genes in various subcategories underwent changes compared to the MPS-Cd results. These results were also observed in the “cellular components” and “molecular functions”, as shown below.

In the “cellular components” (Figure 4b), the DEGs in the “Control vs. MPS group” were divided into 55 subcategories. Many genes in the “cellular components” group had potential functions related to “cell” (54.7%) and “cell part” (54.7%). When comparing MPS with MPS-Cd, the DEGs were grouped into 17 subcategories and related to “cell” (66.7%), “cell part” (66.7%), “organelle” (63%), and “organelle part” (40.7%). In the MPS vs. MPS-Cu group, the DEGs were divided into 222 subcategories, and the most frequently matched genes were related to “cell” (59.3%), “cell part” (59.3%), “organelle” (48.3%), and “organelle part” (31%). Through these results, it can be observed that the DEGs affected by heavy metal exposure are involved in the biological processes of “cell” and “cell part”.

In the “molecular functions” (Figure 4c), the DEGs in the “Control vs. MPS group” were divided into 61 subcategories. Among the genes in the “molecular functions” group, over one-half of the encoded proteins had the function of “catalytic activity” (56.6%) and “binding” (54.7%). When comparing MPS with MPS-Cd, the DEGs were grouped into 27 subcategories and related to “catalytic activity” (77.8%) and “binding” (51.9%). In the MPS vs. MPS-Cu group, the DEGs were divided into 298 subcategories, the most frequently matched genes were related to “Binding” (45.5%) and “catalytic activity” (42.8%). Through these results, it can be observed that the DEGs affected by heavy metal exposure are involved in the biological processes of “catalytic activity” and “binding”.

### 3.6. KEGG Pathway Analysis

To identify the functional implications of the DEGs in *U. pertusa*, we performed pathway analyses using the KEGG database. The results revealed distinct patterns among the different groups (Table 2). In the Control vs. MPS group (Table 2a), the predominant pathway category was associated with membrane-related functions, including mitochondrion inner membrane and membrane protein insertase (OXA1/ALB3/YidC) family, as well as transmembrane region, membrane, and transporter functions. In the MPS vs. MPS-Cd group (Table 2b), one pathway related to transport was consistent with the Control vs. MPS group, while other pathways were primarily linked to oxidative stress, such as oxidoreductase and flavoprotein. Notably, the MPS vs. MPS-Cu group (Table 2c) exhibited similarities with the other groups in terms of transporter pathways but also included unique pathways related to chlorophyll (Chloroplast), binding (ATP-binding, Nucleotide-binding, RNA-binding), organelles (Cytoplasm, Chromosome, Golgi apparatus, Lipoprotein), and carbon metabolism (Carbohydrate metabolism, Pyruvate metabolism). 

### 3.7. Effects of Heavy Metals on Photosynthetic Activity

**Figure 5:**
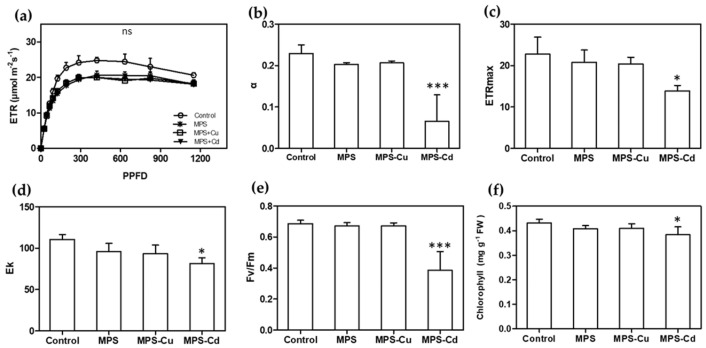
Effects of heavy metals treatment on photosynthesis. Various photosynthetic parameters including RLC (**a**), α (**b**), ETRmax (**c**), E_k_ (**d**), F_v_/F_m_ (**e**), and total chlorophyll content (**f**) were compared between different treatment groups. All data are presented as the mean ± SD (n = 5). Statistically significant differences compared to control are represented as ns; no significant, * *p* < 0.05 or *** *p* < 0.001 above the bar plot.

The RLC can be used to interpret the photosynthetic responses of plants to a range of light levels. Figure 5a shows the ETR, with a linear increase until the point of light limitation, followed by a plateau where the photosynthetic rate was limited. The ETR values also decreased with the heavy metal treatment. In *U. pertusa*, the maximum efficiency of photosystem II, electron transport rate, alpha, minimum fluorescence, maximum electron transport rate, and E_k_ were affected by heavy metal stress. MPS, MPS-Cu, and MPS-Cd treatments caused significant reductions in ETR. In particular, α, ETRmax, E_k_, F_v_/F_m_, and the total chlorophyll content were significantly reduced in the MPS-Cd treatment. α, ETRmax, E_k_, F_v_/F_m_, and chlorophyll content were reduced by approximately 5−16% in the MPS and MPS-Cu treatments compared to those of the control (Figure 5). Heavy metal stress inhibits photosynthetic activity in *Ulva* tissues owing to an imbalance between light input and utilization efficiency. Photosynthetic inhibition occurred in a heavy metal concentration-dependent manner, and ETR decreased at all PPFD levels in response to increased heavy metal stress. 

### 3.8. RT-qPCR

**Figure 6:**
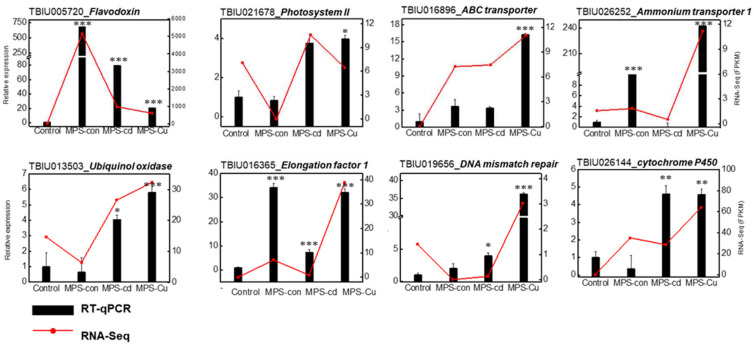
Verification of relative expression levels of DEGs through RT-qPCR. The relative expression values from RT-qPCR are shown as the mean ± SD of the fold-change values from three biological replicates. His2 was used as reference gene for RT-qPCR validation. RT-qPCR was performed on nine heavy metal stress-related genes, including Flavodoxin, Photosystem II, cytochrome P450, Ubiquinol oxidase, Elongation factor1, Ammonium transporter, L-ascorbate peroxidase6, ABC transporter and DNA mismatch repair. All data are presented as the mean ± SD (n = 3). Statistically significant differences compared to the control are represented as * *p* < 0.05 ** *p* < 0.01, *** *p* < 0.001 above the bar plot.

To confirm the difference in the expression of the identified transcripts in *U. pertusa*, eight unigenes were selected for RT-qPCR analysis (Figure 6). Gene expression patterns during heavy metal stress showed upregulation and downregulation of many response genes, such as those encoding “photosynthesis”, “transporter”, “Oxidative stress”, and “DNA repair” (Table 3). The expression levels of genes encoding flavodoxin, photosystem II, cytochrome P450, ubiquinol oxidase, elongation factor1, ammonium transporter, ABC transporter and DNA mismatch repair were significantly elevated in the heavy metal treatments compared to those in the control. Differences in gene expression levels obtained via RNA-seq were similarly represented in the RT-qPCR results.

In the process of selecting reference genes, each gene was assigned specific weights based on their rankings from the RefFinder program, which integrated Delta CT, BestKeeper, Normfinder, and Genorm analyses considering the FPKM and Cq values. By calculating the geometric mean of the assigned weights based on the rankings, His2 was selected as the reference gene for the final overall ranking. (Figure S6).

## 4. Discussion

Heavy metals can affect marine organisms, especially primary producers such as seaweeds, which can accumulate these metals. Thus, the types and concentrations of heavy metals in seawater can have either positive or negative effects on the survival and growth of seaweeds [41]. The aim of this study was to identify the defense mechanisms activated in *U. pertusa* under exposure to different concentrations of heavy metals and obtain information about the genes associated with these mechanisms, as genetic information on *U. pertusa* is currently limited. 

The ocean cannot be completely free from heavy metal contamination. Therefore, each country establishes its standards for the concentration of heavy metals in seawater to manage and prevent adverse effects on marine organisms. South Korea has implemented marine life protection standards to regulate the levels of heavy metal contamination. This study was conducted based on the assumption that concentrations of heavy metals, which comply with marine life protection standards, exist in the seawater. The study categorized the exposure groups into a low-concentration exposure group (MPS), where such concentrations exist, and a high-concentration exposure group (MPS-Cd or MPS-Cu) that assumes situations where sudden accidents or pollution sources introduce high concentrations of cadmium or copper into the seawater.

When *U. pertusa* was exposed to high concentrations of copper and cadmium, its viability was significantly reduced, which is consistent with previous studies that have reported heavy metals inhibiting the growth of *U. pertusa* [28,42]. Interestingly, no significant effect on photosynthetic related parameters, except ETR, were observed in the groups exposed to MPS and MPS-Cu (Figure 5). These results are similar to the finding of Kumar et al. [43], in which the measured values of photosynthetic parameters, except for ETRmax, were not different from the control when exposed to copper at a concentration of 250 μg/L. However, MPS-Cd stress induced decreased photosynthetic performance and ETR. High concentrations of cadmium (>300 μg/L) produce the disturbance in the photosystem II (PSII) related parameters (rETR, *Fν*/*Fm*, and yield) in *Ulva* spp. (*U. rigida* and *U. prolifera*) [16,43,44]. In addition, the gene expression of photosystem II 22 kDa protein, chloroplast (*PSBS*), showed no significant difference between the MPS-treated group and the control group, but was significantly increased by MPS-Cd treatment (Figure 6). 

Previous research has suggested that abiotic stressors, such as cold, light, salt, and metal exposure, can impair the function of photosystems and lead to the production of reactive oxygen species (ROS), with photosystems being the main sites of ROS production and damage [21,28,45,46,47,48]. Interestingly, our study found that despite heavy metal treatment, a set of genes related to photosynthetic activity was upregulated, including one gene from photosystem II (*PSBS*) and one flavodoxin (*FLAV_CHOCR*), which can replace ferredoxin in many reactions (Table 3).

By measuring total chlorophyll concentrations in *Ulva* cells, we confirmed that photosynthesis was inhibited under metal-treated conditions. While MPS and MPS-Cu treatments did not alter the chlorophyll concentration, consistent with previous studies [21,28], MPS-Cd treatment resulted in an approximately 10% decrease in chlorophyll concentration in *U. pertusa* (Figure 5). A recent study by Laporte et al. [21] reported that treatment of *U. compressa* with 10 μM copper (equivalent to 635 μg/L) for 5 days led to an increase in the expression of photosynthesis-related genes, including chlorophyll ab-binding protein, PSII, cytb6f, PSI, and ATP synthetase. Interestingly, our findings using *U. pertusa* showed a similar trend. Our results provide cellular-level evidence supporting the upregulation of photosynthesis-related genes, suggesting that the enhanced photosynthesis-related activities are related to low concentrations of heavy metals and copper tolerance in *U. pertusa*. However, it should be noted that cadmium has a different mechanism of stress response.

**Figure 7:**
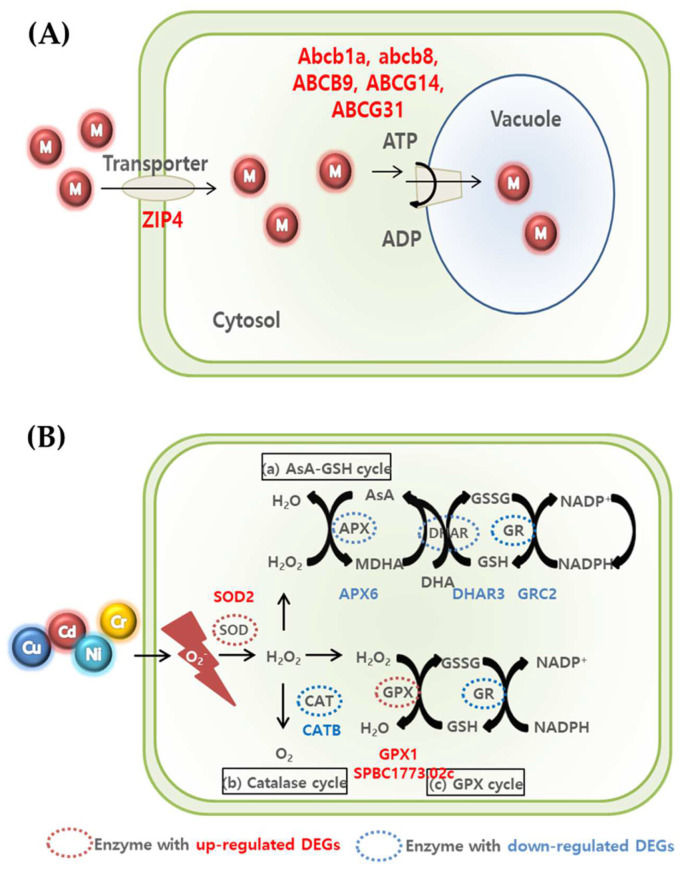
Summary of transporter-related mechanism (**A**) and ROS scavenging (**B**) for metal detoxification and tolerance in *U. pertusa*.

Previous studies have shown that in response to heavy metal exposure, various organisms increase the expression of ABC transporters as a defense mechanism, which can affect growth and viability when inhibited [49,50,51]. In this study, we observed changes in the expression of genes related to ABC transporters of *U. pertusa*. We identified 123 differentially expressed genes encoding ABC transport members in the *U. pertusa*, and of these, ABC transporter genes were significantly and dose dependently upregulated in the heavy metal treatment groups (control vs. MPS, MPS vs. MPS-Cu, and MPS vs. MPS-Cd) (Table 3; Figure 7). Interestingly, the expression levels of ABC transporter genes were upregulated in the MPS group compared to those in the control group but downregulated in the MPS-Cd and MPS-Cu groups compared to those in the MPS group. This suggests that ABC transporter genes may be responsible for low concentrations of heavy metals (Cd or Cu) and regulate the concentration of heavy metal ions in the cell as a defense mechanism against heavy metal stress. Besides ABC transporters, there are also transporters that transport heavy metal ions into or out of cells, such as ZIP4. We found that the expression level of ZIP4 greatly increased in control vs. MPS and MPS vs. MPS-Cu but decreased in MPS vs. MPS-Cd (Table 3). The zip family of transporters may transport Fe^2+^, Mn^2+^, and Zn^2+^ at the plasma membrane or across intracellular membranes of macroalgae, such as *Pyropia* [52]. Moreover, protein plant cadmium resistance 7 (PCR7) is recognized for its function as a cadmium transporter. In particular, a report by Roy et al. [53] indicates that when *Sorghum* (*Sorghum bicolor*) is exposed to cadmium, the expression of this protein decreases. This is consistent with our results, in which reductions were observed in the MPS and MPS-Cd groups.

Heavy metal toxicity is generally associated with ROS, which can induce oxidative stress [17,54]. Although higher plants have been extensively studied for their antioxidant responses to oxidative and environmental stresses, the molecular mechanisms underlying these responses have not been extensively studied in algae. Many plants generate ROS in response to heavy metal exposure, and excessive ROS induces oxidative damage to cellular molecules, leading to cell structure transformation and mutagenesis [55]. ROS production is a major threat to plants, and excessive heavy metal ions can cause oxidative stress in cells by producing the associated oxides. The increase in peroxidase indicates increased stress due to excessive heavy metal ions, destruction of the plasma membrane by lipid peroxidation, and response to ROS produced by excessive heavy metals [56]. Increases in peroxides are caused by reactions mediated by heavy metals [56,57]. For instance, excess heavy metals can limit CO_2_ fixation in the chloroplasts, leading to over-reduction of the photosynthetic electron transport chain, which is a major source of ROS production [58].

Three ways are suggested to remove ROS produced in cells (Figure 7) [59,60,61]. First, (a) the AsA-GSH cycle involves *APX*, *DHAR*, and GR-related genes in the conversion of H_2_O_2_ to water [61]. As a result of this experiment, we confirmed that *APX6, DHAR3,* and *GRC2*, which are related genes, decreased in all the groups, although there was a difference according to the concentration. Second, the expression of *CATB*, which is related in the catalase cycle that H_2_O_2_ is converted to O_2_, also decreased in MPS and MPS-Cu groups. In contrast, the expression of the *CATB* gene exhibited similar behavior in both the MPS-Cd treatment group and the control group. This suggests that the catalase cycle may operate differently in the defense mechanisms against cadmium and copper. Finally, in (c), the GPX cycle is regulated by GPX and GR-related genes. The increase in *GPX1* and *SPBC1773.02c* genes in this experiment indicates that the anti-oxidative mechanism proceeds to remove ROS. In the case of the MPS group, compared with the control group, the expression of *SPBC1773.02c*, *GPX1*, and *SOD2* was upregulated, while the expression of *APX6*, *DHAR3*, and *CATB* was downregulated. Compared with the MPS group, the expression of *SPBC1773.02c*, *SOD2*, and *CATB* was relatively higher in the MPS-Cu group. In contrast, *SPBC1773.02c*, *GPX1*, *SOD2*, and *APX6* genes further decreased in the MPS-Cd group compared to those in the MPS group. This suggests that the low level of heavy metal stress at the MPS level is also highly related to oxidative stress and that the defense mechanism is activated. Intracellular ROS is known to cause DNA damage by fermenting lipids and nucleic acids. Therefore, the expression of the genes involved in DNA repair is expected to increase. As shown in Table 3, two genes (*MSH5* and *Rad51c*) involved in DNA repair were also expressed. 

In addition, the mitochondria and chloroplasts are cell compartments that are very sensitive to oxidative damage due to the strong electron flux in the microenvironment, including increased oxygen and high metal ion concentrations [12]. The expression of chlorophyll-related genes (*CAB*, *CAB-151*, *CAB1R*, *CAB5*, and *Cabll-1*) was reduced in the group exposed to high copper concentrations (MPS vs. MPS-Cu), indicating that copper ions have a particular impact on the chlorophyll-related mechanisms [19,21]. Diatoms exposed to copper showed increased carbohydrate production [62]. Excessive concentrations of copper and cadmium in the cell leads to significant damage to the cellular process, such as the inhibition of plant growth and photosynthetic electron transfer, resulting in reduced biomass and chlorophyll-related mechanisms (Figure 6) [13,14]. The *TAP46* gene, which is essential for plant growth [63], was significantly downregulated in this study. High concentrations of copper and cadmium inhibited plant growth by decreasing *TAP46* expression (Table 3).

In conclusion, this study has provided insights into the potential involvement of various stress defense mechanisms in *U. pertusa* exposed to both low and high concentrations of copper and cadmium at the transcriptomic level. These defense mechanisms may be primarily associated with photosynthesis, antioxidant mechanisms, ion transporters, and growth. Furthermore, by conducting a transcriptomic study of *U. pertusa* and comparing it with other similar *Ulva* species, we identified the relevant genes and found that the *Ulva* species appropriately utilize defense mechanisms against abiotic stressors, such as heavy metals, to survive under various types of environmental stress. This study provides valuable information about the mechanisms that enable *Ulva* species to adapt to challenging environments and can serve as a basis for analyzing the causes of green tide caused by *U. pertusa* and developing strategies to mitigate them in the future.

## Figures and Tables

**Table 1:** Summary of transcriptome assembly and unigenes.

	No. Unigenes
Number of contigs	60,909
Unigenes base	57,339,822
Average (bp)	941
Minimum length (bp)	201
Maximum length (bp)	17.696
N50 contig length (bp)	1.594
GC (%)	54.5

**Table 2:** Significantly enriched pathways of differentially expressed unigenes from the (**a**) Con vs. MPS, (**b**) MPS vs. MPS-Cd and (**c**) MPS vs. MPS-Cu.

(a)				
Pathway Term	Count of DEGs	*p*-Value	Benjamini	FDR
Transit peptide	6	1.10 × 10^−3^	4.80 × 10^−2^	0.05
Peptide Mitochondria	4	2.90 × 10^−3^	1.30 × 10^−1^	0.13
Mitochondrion inner membrane	3	3.60 × 10^−3^	7.80 × 10^−2^	0.08
Membrane insertase OXA1/ALB3/YidC	2	4.10 × 10^−3^	1.10 × 10^−1^	0.17
Mitochondria	4	7.90 × 10^−3^	3.30 × 10^−1^	7.19
Transmembrane region	6	1.60 × 10^−2^	3.30 × 10^−1^	0.33
ABC-2 type transporter	2	2.40 × 10^−2^	4.30 × 10^−1^	0.43
Protein export	2	5.30 × 10^−2^	3.50 × 10^−1^	25.87
ABC transporter-like	2	6.80 × 10^−2^	6.60 × 10^−1^	48.5
Membrane	7	7.10 × 10^−2^	5.70 × 10^−1^	0.57
**(b)**				
**Pathway Term**	**Count of DEGs**	***p*-Value**	**Benjamini**	**FDR**
Oxidoreductase	3	3.70 × 10^−2^	8.20 × 10^−1^	29.95
Flavoprotein	2	5.90 × 10^−2^	7.40 × 10^−1^	43.33
Transit peptide	3	7.50 × 10^−2^	6.90 × 10^−1^	52.12
**(c)**				
**Pathway Term**	**Count of DEGs**	***p*-Value**	**Benjamini**	**FDR**
Chloroplast	25	7.30 × 10^−10^	1.10 × 10^−7^	8.77 × 10^−7^
Pastid	25	8.00 × 10^−10^	6.00 × 10^−8^	9.52 × 10^−7^
Transit peptide	23	7.30 × 10^−7^	3.70 × 10^−5^	8.75 × 10^−4^
ATP-binding	23	2.50 × 10^−6^	9.60 × 10^−5^	3.03 × 10^−3^
Nucleotide-binding	24	7.90 × 10^−6^	2.40 × 10^−4^	9.39 × 10^−3^
phosphoprotein	19	2.10 × 10^−5^	4.60 × 10^−4^	0.03
Metal-binding	26	5.50 × 10^−5^	1.00 × 10^−3^	0.01
Transferase	27	8.90 × 10^−5^	1.50 × 10^−3^	0.01
Alternative splicing	25	2.60 × 10^−4^	3.90 × 10^−3^	0.31
RNA-binding	9	1.20 × 10^−3^	1.50 × 10^−2^	1.47
Carbohydrate metabolism	5	2.40 × 10^−3^	2.70 × 10^−2^	2.8
Chromatin regulator	5	3.70 × 10^−3^	3.90 × 10^−2^	4.32
Chromosome	4	1.00 × 10^−2^	1.00 × 10^−1^	11.76
Cytoplasm	14	1.10 × 10^−2^	9.36 × 10	11.99
Pyruvate metabolism	3	1.70 × 10^−2^	1.40 × 10^−1^	18.34
Serine/threonine-protein kinase	8	2.00 × 10^−2^	1.60 × 10^−1^	21.86
Ribosomal protein	6	2.10 × 10^−2^	1.50 × 10^−1^	21.96
Meiosis	3	2.10 × 10^−2^	1.50 × 10^−1^	22.7
Magnesium	7	2.30 × 10^−2^	1.50 × 10^−1^	24.14
Zinc	11	2.30 × 10^−2^	1.50× 10^−1^	24.5
Zinc-finger	9	5.60 × 10^−2^	1.60 × 10^−1^	27.29
Cell division	4	3.40 × 10^−2^	1.90 × 10^−1^	33.68
Golgi apparatus	6	4.10 × 10^−2^	2.20 × 10^−1^	39.5
Myristate	3	4.50 × 10^−2^	2.40 × 10^−1^	42.57
Ribonucleoprotein	6	4.80 × 10^−2^	2.40 × 10^−1^	44.23
Cell cycle	4	6.30 × 10^−2^	2.90 × 10^−1^	53.85
Lipoprotein	5	7.40 × 10^−2^	3.30 × 10^−1^	59.89
Nucleus	19	8.30 × 10^−2^	3.40 × 10^−1^	64.42
Protein kinase, ATP binding site	7	8.10 × 10^−2^	8.80 × 10^−1^	67.86
Galactose metabolism	3	8.30 × 10^−2^	8.70 × 10^−1^	56.39
Histidine phosphatase superfamily, clade-1	2	9.40 × 10^−2^	8.60 × 10^−1^	72.8
RNA recognition motif domain, eukaryote	2	9.80 × 10^−2^	8.50 × 10^−1^	74.36
DNA repair	3	1.00 × 10^−1^	3.80 × 10^−1^	71.48
ABC transporter	3	1.00 × 10^−1^	8.90 × 10^−1^	74.92

**Table 3:** Gene expression levels (FPKM) of chlorophyll, photosynthesis, transporter, oxidative stress, DNA repair, and growth-related genes for each treatment group.

GeneID	Gene Name	Description	Length	Sample (FPKM)				Control vs. MPS		MPS vs. MPS-Cd		MPS vs. MPS-Cu	
				Control	MPS	MPS-Cd	MPS-Cu	Log2(Fold Change)	*p*-Value	Log2(Fold Change)	*p*-Value	Log2(Fold Change)	*p*-Value
***Chlorophyll related gene***													
TBIU006309	*CAB5*	Chlorophyll a-b binding protein 5, chloroplastic	340	3684.33	7709.60	100.00	1429.17	1.01	0.7574	−6.57	0.1165	−2.64	0.0052
TBIU009506	*CAB-151*	Chlorophyll a-b binding protein 151, chloroplastic	1361	422.18	11,305.50	176.07	1815.70	4.71	0.1992	−6.34	0.1266	−2.86	0.0026
TBIU009507	*cabII-1*	Chlorophyll a-b binding protein of LHCII type I, chloroplastic	1166	1341.70	3696.57	29.81	595.29	1.43	0.6653	−7.29	0.0892	−2.85	0.0027
TBIU018946	*CAB1R*	Chlorophyll a-b binding protein 1, chloroplastic	2117	9.43	1190.41	124.78	187.39	6.95	0.0818	−3.59	0.3414	−2.88	0.0025
TBIU018948	*CAB*	Chlorophyll a-b binding protein type 2 member 2	581	1900.00	4920.06	71.41	784.77	1.31	0.6905	−6.38	0.1252	−2.86	0.0029
***Photosynthesis***													
TBIU005720	*FLAV_CHOCR*	Flavodoxin	1050	13.32	5149.3	978.56	606.5	8.56	0.0426	−2.73	0.4576	−3.30	0.0006
TBIU021678	*PSBS*	Photosystem II 22 kDa protein, chloroplastic	1249	7.07	0	10.56	6.47	−13.20	0.1094	13.40	0.0995	12.80	0.0021
***Transporter***													
TBIU016896	*ABCG14*	ABC transporter G family member 14	1520	0.01	7.23	7.44	10.98	10.10	0.0353	−0.30	0.9309	0.38	0.6775
TBIU026252	*AMT1-1*	Ammonium transporter 1 member 1	757	1.57	1.84	0.51	11.21	0.20	0.9608	−2.18	0.5711	2.39	0.0353
TBIU004363	*AMT1-5*	Putative ammonium transporter 1 member 5	724	47.91	2.09	0.06	105.31	−4.55	0.2154	−5.55	0.2160	5.44	0.0000
TBIU019707	*At3g26670*	Probable magnesium transporter NIPA8	930	2.37	0.04	0.05	3.55	−5.77	0.1754	−0.25	1.0000	6.11	0.0003
TBIU001354	*ATX1*	Copper transport protein ATX1	212	0.51	52.23	17.3	107.9	6.63	0.1098	−1.92	0.5966	0.831	0.3847
TBIU001726	*Atp7a*	Copper-transporting ATPase 1	1138	0.45	2.57	1.00	4.99	2.46	0.7163	−1.67	0.7209	0.743	0.7853
TBIU016009	*HMA5*	Probable copper-transporting ATPase HMA5	495	0.31	5.46	2.20	13.11	4.11	0.3310	−1.64	0.6601	1.05	0.3805
TBIU016264	*ZIP4*	zinc transporter 4, chloroplastic	469	0.00	4.74	1.91	13.71	10.70	0.3977	−1.62	0.7033	1.32	0.0428
TBIU032918	*PCR7*	Protein PLANT CADMIUM RESISTANCE 7	2459	18.7	1.09	0.29	41.51	−4.13	0.2643	−2.25	0.5875	5.03	0.0000
TBIU026011	*SFC1*	Mitochondrial succinate-fumarate transporter 1	1181	1.44	3.84	6.60	0.66	1.39	0.6850	0.44	0.9032	−2.77	0.0209
***Oxidative stress***													
TBIU026067	*PHGPx*	Probable phospholipid hydroperoxide glutathione peroxidase	1103	1.01	0.00	1.32	2.47	−12.60	0.1561	12.60	0.1406	13.60	0.0001
TBIU015022	*CATB*	Catalase isozyme B	524	8.92	1.84	10.82	3.04	−2.31	0.5019	2.22	0.5445	0.509	0.6648
TBIU019894	*SPBC1773.02c*	Peroxiredoxin C1773.02c	1381	0.74	18.91	5.95	110.58	4.65	0.2460	−2.00	0.5856	2.33	0.0197
TBIU051511	*GPX1*	Glutathione peroxidase	377	2.20	5.84	2.86	4.24	1.36	0.7824	−1.34	0.7509	−0.68	0.7740
TBIU020456	*SOD2*	Superoxide dismutase [Mn]	302	5.82	66.65	23.85	235.55	3.48	0.3317	−1.81	0.6165	1.61	0.0889
TBIU007783	*DHAR3*	Glutathione S-transferase DHAR3, chloroplastic	389	327.98	165.24	182.15	137.00	−1.02	0.7555	−0.20	0.9543	−0.49	0.5882
TBIU043094	*APX6*	Probable L-ascorbate peroxidase 6, chloroplastic	207	248.46	181.55	61.84	172.26	−0.49	0.8818	−1.89	0.5997	−0.29	0.7447
TBIU022271	*GRC2*	Glutathione reductase, cytosolic	980	370.65	152.22	285.81	109.19	−1.32	0.6900	0.57	0.8735	−0.70	0.4380
***DNA repair***													
TBIU019656	*MSH5*	DNA mismatch repair protein MSH5	1300	1.40	0.00	0.13	3.02	−11.90	0.2216	8.10	0.7450	12.80	0.0028
TBIU026428	*Rad51c*	DNA repair protein RAD51 homolog 3	926	17.94	6.84	5.30	35.73	−1.42	0.6709	−0.71	0.8429	2.17	0.0265
***Growth***													
TBIU021761	*TAP46*	PP2A regulatory subunit TAP46	1427	0.00	3.02	7.30	6.12	15.00	0.0467	0.93	0.7953	0.80	0.4086

## Data Availability

The data presented in this study are available in Supplementary Material (Figures S1–S5; Tables S1–S4).

## Supplementary Materials

The following supporting information can be downloaded at: https://www.mdpi.com/article/10.3390/toxics11070549/s1, Figure S1: Schematic showing the treatment protocol of low and high concentrations of heavy metals in *Ulva pertusa*; Figure S2: Length frequency distribution of assembled unigenes; Figure S3: A total of 60,909 unigenes were queried against the nonredundant (nr) protein database; Figure S4: Taxonomy of *U. pertusa* using Krona; Figure S5: Scatter plot of FPKM values between pairs of samples. The red dot means up-regulated expression, and the blue means down-regulated expression. Pearson correlation coefficient was used to compare gene expression levels between samples; Figure S6: Expression stability ranking of the nine candidate reference genes evaluated by RefFinder in *U. pertusa.* The ranking of the nine candidate reference genes was based on a combined analysis of gene expression in the Con, MPS, MPS-Cu, and MPS-Cd conditions. Table S1: Marine ecosystem protection standards of Republic of Korea for determining heavy metal treatment concentration; Table S2: List of primers used in RT-qPCR for the validation of DEGs from this study; Table S3: List of candidate reference genes which are stably expressed in *U. pertusa*; Table S4: Summary of RNA-seq results for four treatment groups.
