# Supplementary material for: Transcriptomic Changes Induced by Low and High Concentrations of Heavy Metal Exposure in Ulva pertusa
Source: Toxics. 2023 Jun 22;11(7):549. doi: 10.3390/toxics11070549 (PMC10383703; doi:10.3390/toxics11070549)
Supplement: Supplementary file 1 [file toxics-11-00549-s001.zip › toxics-2397794-supplementary.pdf]

Table S1. Marine ecosystem protection standards of Republic of Korea for determining heavy metal treatment concentration. In the experiment, the values expressed in bold letters was chosen as MPS treatment group.

(Unit : μg/L)	**Cu**	**Pb**	**Zn**	**As**	**Cd**	**Cr** $^{6+}$	Hg	**Ni**
**Short-term***	**3**	7.6	34	9.4	**19**	**200**	1.8	**11**
**Long-term****	1.2	1.6	11	3.4	2.1	2.8	1.0	1.8

*Short-term : Compared with one-time observations

**Long-term : Compared with the minimum seasonal survey data

Table S2. List of primers used in RT-qPCR for the validation of DEGs from this study.

Contig ID	Gene description	Primer sequence (Forward /Reverse)	Amplicon size (bp)
TBIU005720	Flavodoxin	GCCATGAGTTCCGATGAGTT<br>CATGGCATCGCAGAAGTAGT	102
TBIU014014	Lectin	CGGTCGTCTTCCTGTTTGTT<br>TGCTGTCCTTCGGGTAGTAT	126
TBIU016896	ABC transporter G family member 14	CGCTCAATCGCTCTCTCAAA<br>GAGGCGTGCTGCATAATAGA	104
TBIU026144	cytochrome P450 CYP13A5	GGTTCCTGCCATTCCTTGA<br>GACGCCACCAGCATCTT	82
TBIU008580	ATP-dependent zinc metalloprotease FTSH 2, chloroplastic	CTCATCCCGCATGTCTTACTC<br>CGATGGTGCCATTCTCGTATAG	89
TBIU013503	Ubiquinol oxidase 4, chloroplastic/chromoplastic	AGCAAGCATACCTGTTCTCC<br>TGGCAGCGACTTCAACAA	107
TBIU016365	Elongation factor 1-alph	TGAGGTTCAAGGCCAAACTC<br>CATGTCGCAGTCCATCTTGT	97
TBIU021678	Photosystem II 22 kDa protein, chloroplastic	CACTACTGCCAGGTGATCTT<br>GCGGCAGAAAGTGTGAATAC	115
TBIU012199	E3 ubiquitin-protein ligase RGLG2	ATCGAAGTACCCGCTATCAATC<br>AAATTGTCCCACTCCCTAGC	105
TBIU026252	Ammonium transporter 1 member 1	TCAGATGGGACTGCTAGTGA<br>GAACCGGGATTGAAGCCATA	104
TBIU012687	Histone H2AX	ATGCCGCGAGAGACAATAAG<br>TCACAGAGCCGAGAAGTTTG	99
TBIU006309	Chlorophyll a-b binding protein 5	GTTCTCCATGTTCGGCTTC<br>AACTTGGTGGCGTAGTTCC	120
TBIU009507	Chlorophyll a-b binding protein of LHCII type I	CTTGGCAACTCTTCCCTCATC<br>GTTGACACGGTATCCCTCAATC	96
TBIU018948	Chlorophyll a-b binding protein type 2	ACTACCTCGGCAACCCTAA<br>TTACGCGGTATCCCTCAATC	120
TBIU028181	ABC transporter G family member 31	TTCACCTACCTCATGCTCTTTG<br>CGGGAATGCTAGAGAGGTACT	103
TBIU026067	phospholipid hydroperoxide glutathione peroxidase	GATAGTACTGCGGGCTTGTT<br>CGACCAGTGAATCCCATATGTC	106
TBIU026428	DNA repair protein RAD51	CTGCGAAGAGGTGACAGAAT<br>CCACATACAGGTGGTAGTTGAG	99

Table S3. List of candidate reference genes which are stably expressed in *U. pertusa.*

Contig ID	Gene symbol	Gene length (bp)	The best BLASTX hit against NCBI nr DB	Control FPKM	MPS FPKM	MPS-Cd FPKM	MPS-Cu FPKM
TBIU002383	H1-II	234	Histone H1-II [Source:SWISS;ACC:Q08865]	37.93	47.73	46.92	59.32
TBIU012687	H2AX_PICAB	1087	Histone H2AX [Source:SWISS;ACC:P35063]	175.77	143.65	200.73	200.6
TBIU012997	H3a	626	Histone H3.3 type a [Source:SWISS;ACC:O15819]	183.62	200.98	268.24	242
TBIU015557	H4_OLILU	1072	Histone H4 [Source:SWISS;ACC:P82888]	214.37	181.26	173.32	173
TBIU020957	H2B4_VOLCA	477	Histone H2B.4 [Source:SWISS;ACC:P16868]	117.74	149.57	236.9	202.3
TBIU002688	TUBA1	1898	Tubulin alpha-1/alpha-2 chain [Source:SWISS;ACC:P11481]	18.66	28.51	92.35	45.72
TBIU009060	TUBD1	315	Tubulin delta chain [Source:SWISS;ACC:Q8HZV4]	21.24	18.17	27.95	25.05
TBIU014457	Wbscr22	1504	Probable 18S rRNA (guanine-N(7))-methyltransferase [Source:SWISS;ACC:Q9CY21]	5.45	5.07	9.1	4.66
TBIU014458	Wbscr22	2064	Probable 18S rRNA (guanine-N(7))-methyltransferase [Source:SWISS;ACC:Q9CY21]	31.7	38.46	45.15	36.47

Table S4. Summary of RNA-seq results for four treatment groups

Library	Raw data			Filtered data		
	# Reads[1]	Total Bases[2]	Base (>Q30)[3]	# Reads[1]	Total Bases[2]	trimmed[4]
**Control**	61,173,540	6,178,527,540	5,672,919,713	55,835,312 (91.30%)	5,625,425,236 (91.00%)	56,063,064 (91.60%)
**MPS**	62,244,430	6,286,687,430	5,754,207,889	56,533,282 (90.80%)	5,695,385,854 (90.60%)	56,794,734 (91.20%)
**MPS-Cd**	71,130,802	7,184,211,002	6,590,337,115	64,698,188 (91.00%)	6,515,000,835 (90.70%)	62,035,670 (91.40%)
**MPS-Cu**	67,608,248	6,828,433,048	6,244,062,288	61,070,548 (90.30%)	6,149,791,570 (90.10%)	61,408,464 (90.80%)

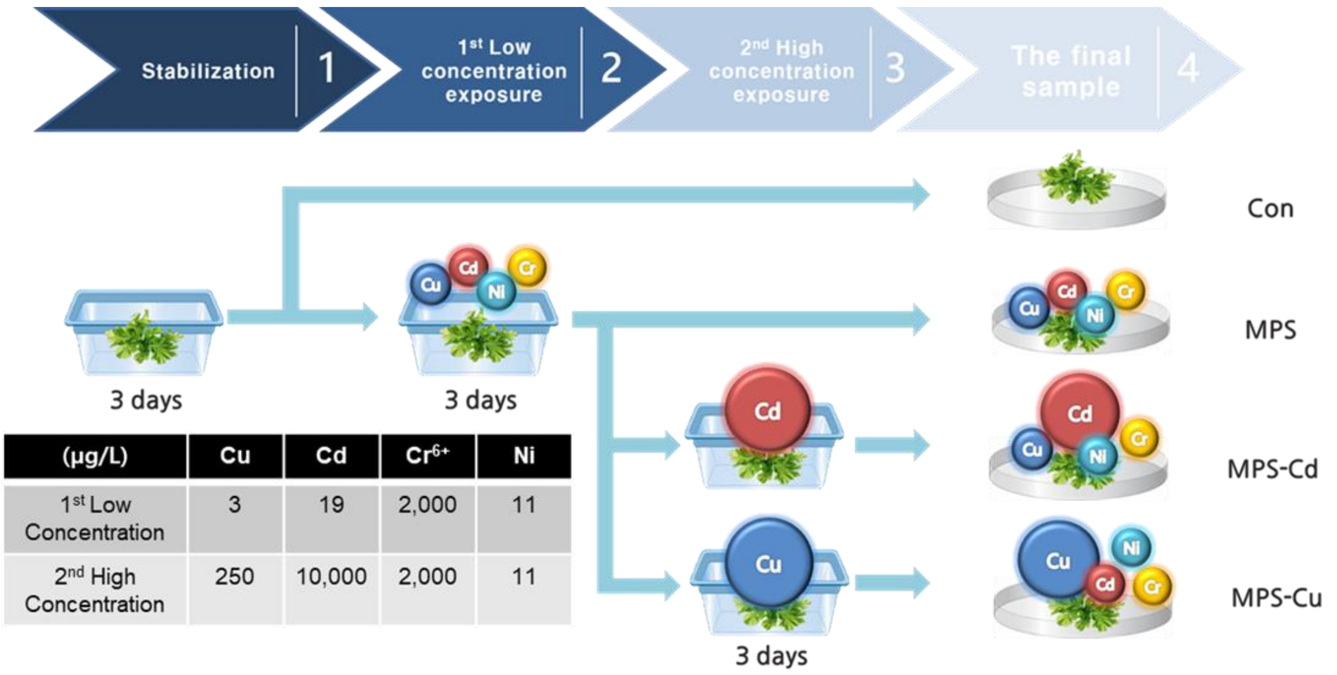

(μg/L)	Cu	Cd	Cr^{6+}	Ni
1st Low Concentration	3	19	2,000	11
2nd High Concentration	250	10,000	2,000	11

Figure S1. Schematic showing the treatment protocol of low and high concentrations of heavy metals in *Ulva pertusa*.

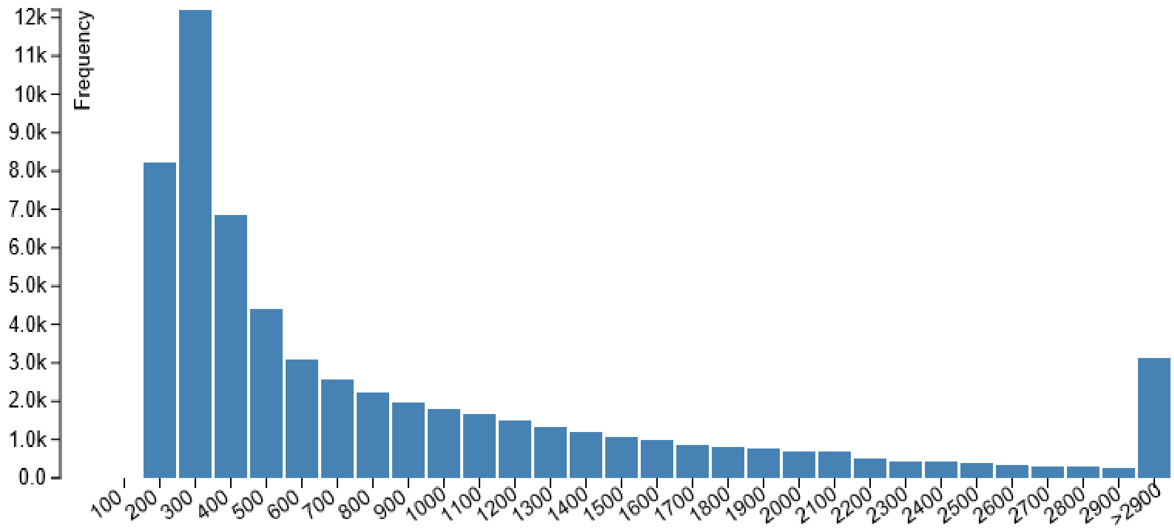

Figure S2. Length frequency distribution of assembled unigenes.

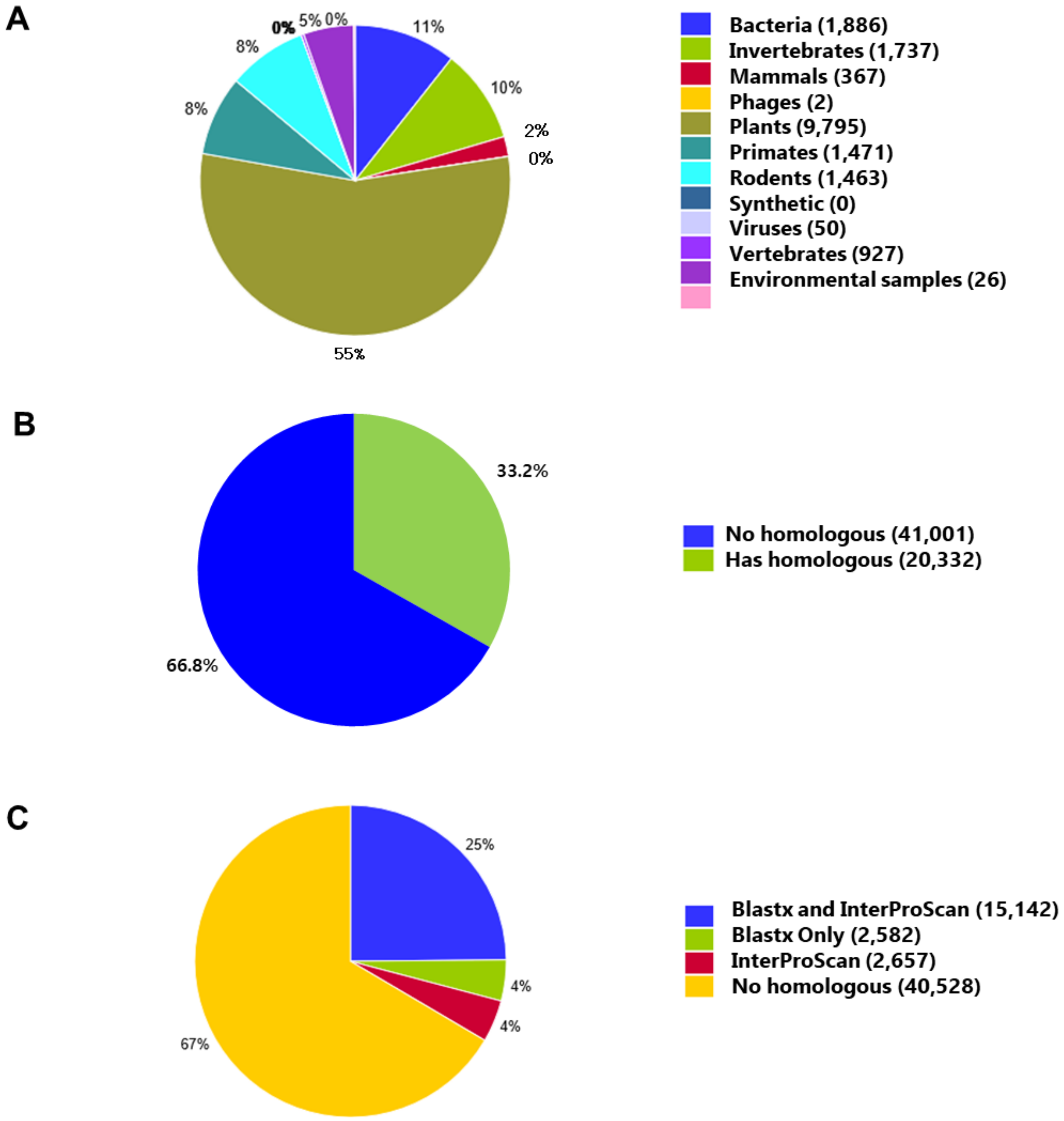

Figure S3. A total of 60,909 unigenes were queried against the nonredundant (nr) protein database. (A) DNA sequence-based homology search using Blastx, (B) Protein-based homology search using InterProScan, (C) Consensus between Blastx and InterProScan (cut off: E-value > 10^{-5}).

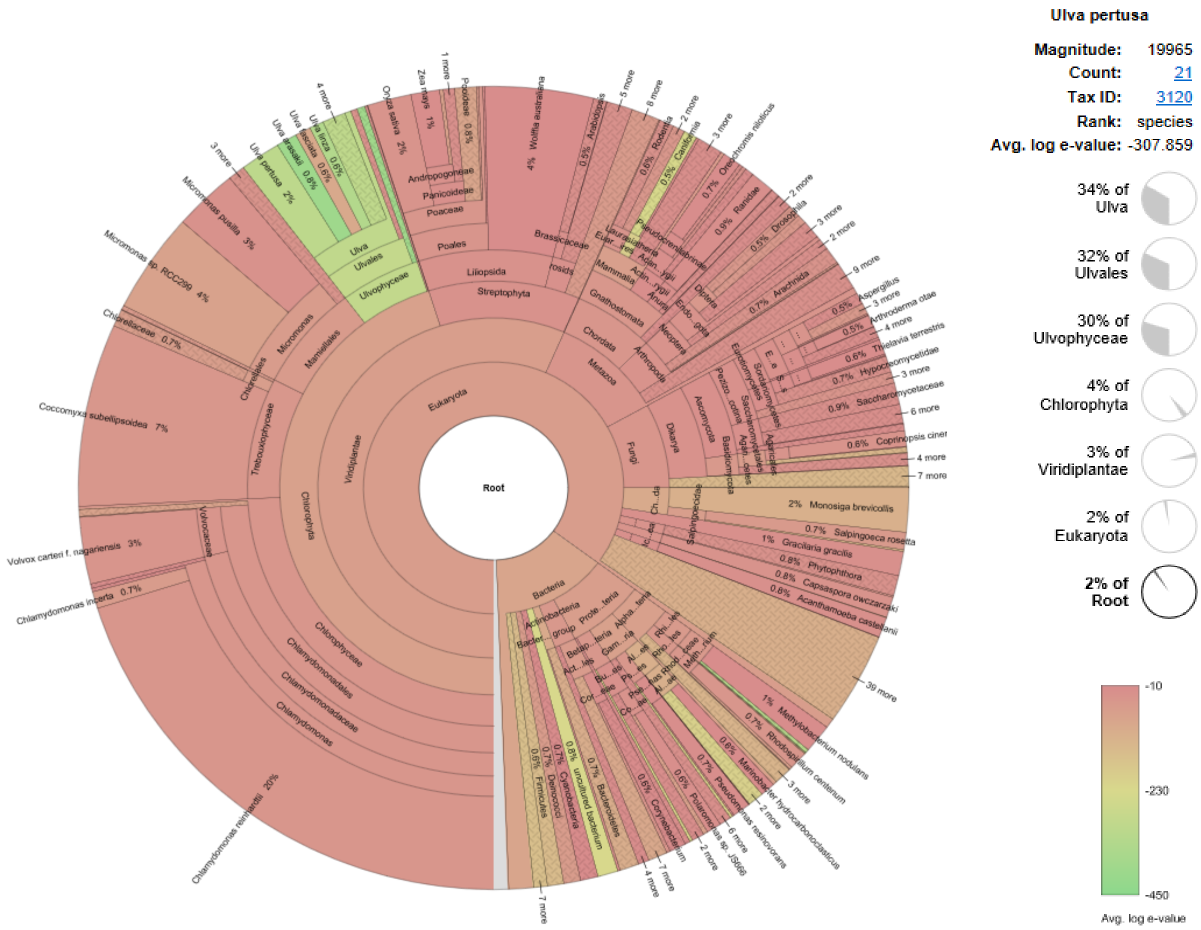

Figure S4. Taxonomy of *U. pertusa* using Krona.

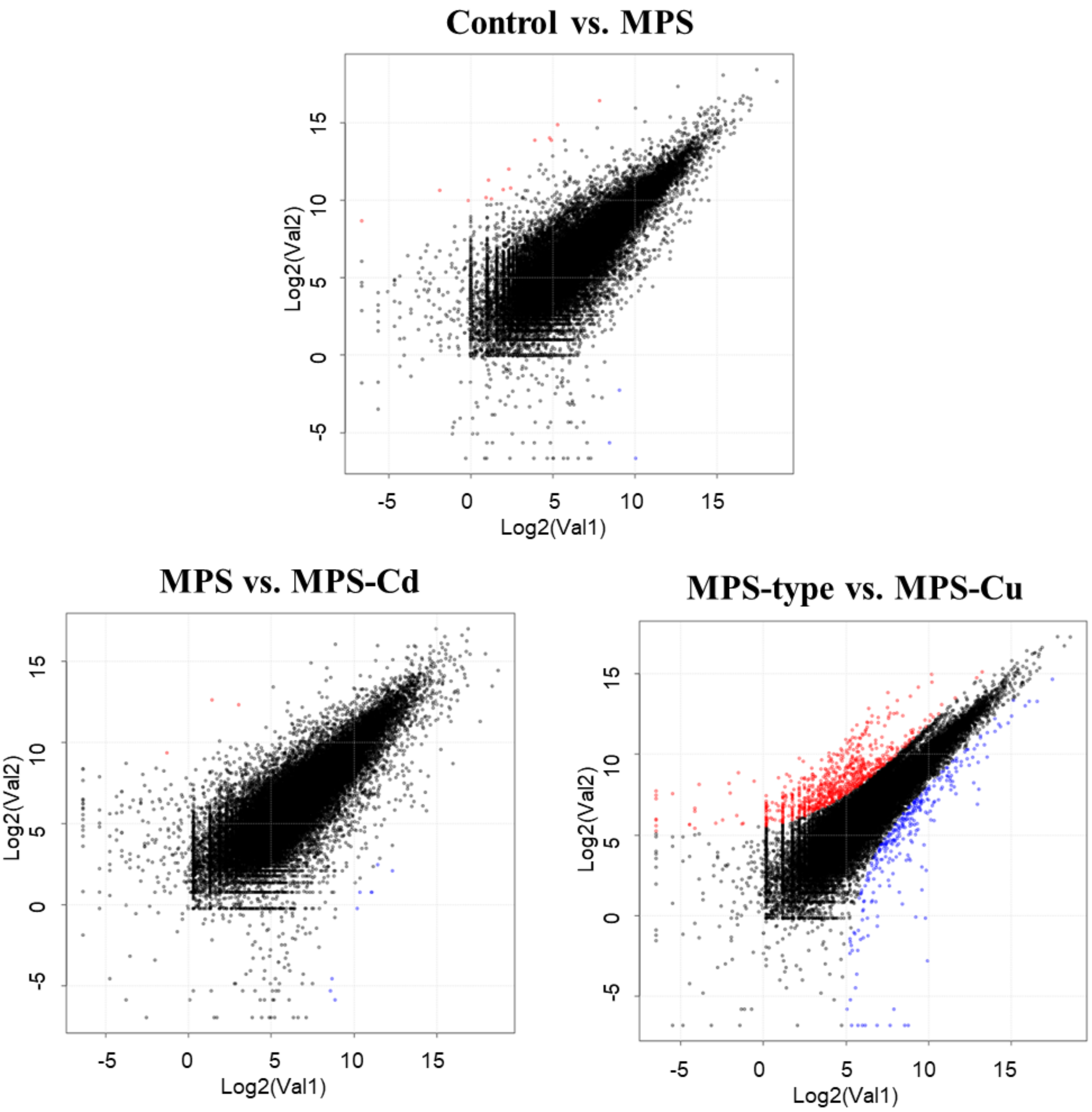

Figure S5. Scatter plot of FPKM values between pairs of samples. The red dot means up-regulated expression, and the blue means down-regulated expression. Pearson correlation coefficient was used to compare gene expression levels between samples.

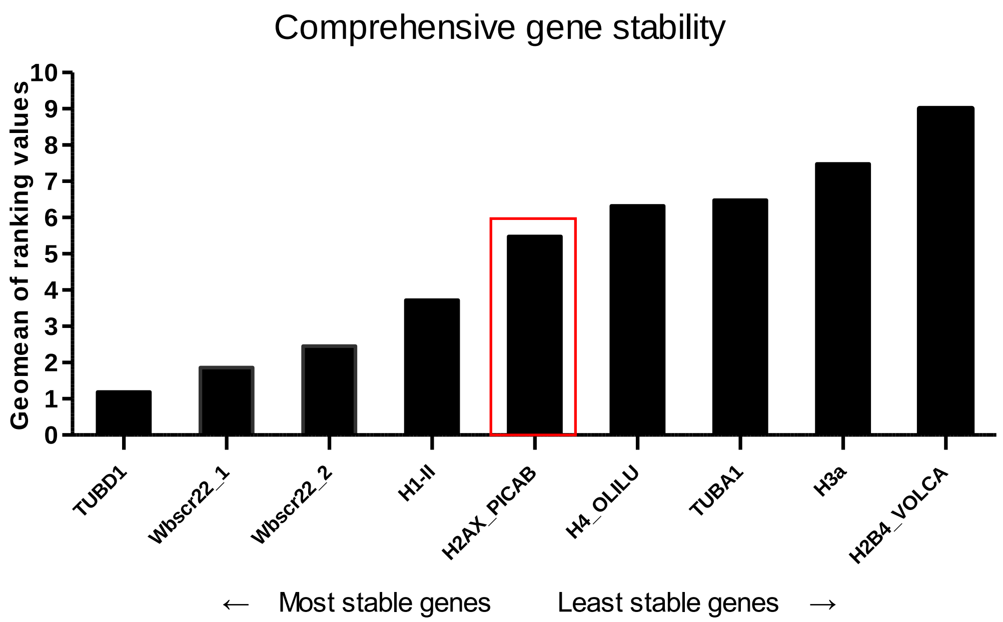

Figure S6. Expression stability ranking of the nine candidate reference genes evaluated by RefFinder in *U. pertusa.* The ranking of the nine candidate reference genes was based on a combined analysis of gene expression in the Con, MPS, MPS-Cu, and MPS-Cd conditions.
